# Cone Beam Online Adaptive Radiation Therapy: A Promising Approach for Gastric Mucosa-Associated Lymphoid Tissue Lymphoma?

**DOI:** 10.1016/j.adro.2024.101692

**Published:** 2024-12-02

**Authors:** Xinran Zhong, Mahbubur Rahman, Ambrosia Simmons, Xingzhe Li, Malgorzata Kozak, Neil Desai, Robert Timmerman, Andrew Godley, Bin Cai, David Parsons, Kiran A. Kumar, Mu-Han Lin

**Affiliations:** Department of Radiation Oncology, UT Southwestern Medical Center, Dallas, Texas

## Abstract

**Purpose:**

Daily online adaptive radiation therapy (oART) opens the opportunity to treat gastric mucosa-associated lymphoid tissue (MALT) lymphoma with a reduced margin. This study reports our early experience of cone beam computed tomography (CBCT)-based daily oART treating gastric MALT lymphoma with breath-hold and reduced margins.

**Methods and Materials:**

Ten patients were treated on a CBCT-based oART system. Organs at risk (OARs) and the clinical target volume (CTV) were adjusted based on the daily CBCT. Planning target volume (PTV) was derived from the CTV with a 0.5 to 0.7 cm margin with breath-hold. Multiple beam arrangements were compared during the preplanning phase to ensure minimal monitor unit (MU) for patient comfort and breath-hold reproducibility. For 108 fractions from the 10 patients, the PTV, CTV coverage, and Paddick conformity index (CI) were compared between the adapted and scheduled plans. The MU, Paddick CI, and gradient index were compared using relative percentage differences between the adapted plans and preplans. The OAR doses from 106 fractions across 9 patients were reported for the preplans, adapted plans, and scheduled plans. The time statistics for each step of the clinical workflow were recorded and reported for 93 treatment fractions from 9 patients.

**Results:**

The PTV volume varied from −37.1% to 90.5% (11.7% ± 18.5%) throughout treatments across all patients. The adapted plan was chosen as the treatment plan for each fraction because of superior PTV and CTV coverage while maintaining a similar OAR dose. The PTV and CTV coverage for the adapted and scheduled plans was V_Rx_ = 95.0% ± 0.3% versus 64.1 ± 19.6% and V_Rx_ = 99.9 ± 0.1% versus 74.0% ± 22.2%, respectively. The adapted plans’ MU, Paddick CI, and gradient index were, on average, 4.1%, 0.4%, and −4.2% of the preplan values, respectively. The console's adaptive workflow and physician time were 25 ± 7 and 19 ± 6 minutes, respectively.

**Conclusion:**

A CBCT-based oART system with the proposed workflow is feasible for treating patients with gastric MALT lymphoma using a reduced PTV margin while maintaining excellent target coverage within a reasonable time, resulting in consistent adapted plan quality. This approach can be expanded to a larger cohort of gastrointestinal patients.

## Introduction

Gastric mucosa-associated lymphoid tissue (MALT) lymphoma is a common type of MALT lymphoma, for which radiation therapy (RT) has been reported to be highly effective for local control.^1^^,^^2^ Thus, reducing long-term toxicity is essential for this group of patients. The surrounding important organs at risk (OARs) include the heart, kidneys, spleen, liver, and bowel. With conventional image guided RT (IGRT), the margin is usually 1.5 to 2.5 cm to account for the large intrafraction and interfraction motion of the stomach.^3^, ^4^, ^5^ With the development of online adaptive RT (oART) systems to address interfraction motion, the margin can be reduced, thereby decreasing the OAR dose further.^6^

Recently, magnetic resonance guided online adaptive RT (MRgRT) has been at the forefront of using oART to treat gastric MALT lymphoma with reduced planning target volume (PTV) margins (0.5-0.7 cm expansion from clinical target volume [CTV]).^7^, ^8^, ^9^ MRgRT takes advantage of improved soft tissue contrast, and 1 institution uses artificial intelligence (AI) techniques for volume delineation.^7^ MRgRT with reduced margins provided adapted plans with improved target coverage while maintaining OAR doses. Despite superior image quality, MRgRT systems are not widely available or adopted.

Cone beam computed tomography (CBCT) guided oART can serve as an alternative to MRgRT, offering daily adapted treatments with reduced margins. However, there are potential challenges to using CBCT guided oART, including imaging artifacts, target visibility, breathing motion management, adaptation robustness, and the extra resources required compared to IGRT. Ethos (Varian Medical Systems) is a CBCT-based oART system reported to be able to treat tumors across different sites, including the abdomen and pelvis.^10^, ^11^, ^12^, ^13^, ^14^, ^15^, ^16^ However, most studies on the abdomen are emulation-based feasibility studies.^13^^,^^16^ Additionally, no studies have investigated using Ethos for treating MALT lymphoma, which presents significant target size and shape variation across fractions with conventional fractionation. It is hypothesized that the Ethos system could effectively treat MALT lymphoma patients with sufficient target coverage with reduced margins within a reasonable timeframe.

This study is the first to report on the utilization of a CBCT guided daily oART system, Ethos, for treating gastric MALT lymphoma with breath-hold and reduced margins. Active Breathing Coordinate (ABC) (Elekta Oncology Systems Ltd) or surface guided RT (SGRT) (AlignRT InBore, AlignRT, Vision RT)-based deep inspiration breath-hold (DIBH) was used to manage motion, with a 0.5 to 0.7 cm PTV margin accounting for intrafraction motion. A robust planning approach was developed to ensure the quality of adapted plans, emphasizing their resilience and efficacy. By analyzing 108 treated adaptive fractions, we demonstrated the feasibility of our workflow, the dosimetric advantages of adapted plans, and the efficiency of CBCT guided oART.

## Materials and Methods

### Patient cohort

With institutional review board approval, ten consecutive patients diagnosed with gastric B-cell lymphoma treated from May 2022 to April 2024 were retrospectively included in this study. All patients were diagnosed with gastric MALT lymphoma. Nine patients received 24 Gy in 12 or 16 fractions, and 1 patient received 4 Gy in 2 fractions. A total of 108 fractions from these 10 patients were included in the data analysis. The patient treated with 4 Gy was excluded from the OAR dose evaluation, and the remaining data analysis was independent of the prescription dose.

### Motion management and treatment workflow

Either ABC or SGRT guided DIBH was used during both simulations and treatments. The first 3 patients used ABC, and the rest of the patients used SGRT guided DIBH. Three breath-hold scans were acquired during the simulation to evaluate the breath-hold consistency. DIBH was used to reduce intrafraction motion, improve CBCT imaging quality during adaptation, and increase the distance between the heart and the target. All 10 patients were tested to perform a 30-second breath-hold during the simulation and did 30-second breath-hold sessions throughout each treatment. The CTV was defined as the gross tumor volume, when visible, and the whole stomach volume from the gastroesophageal junction to beyond the duodenal bulb, including the entire stomach wall and visible perigastric nodes.^3^ A PTV margin of 5 to 7 mm from the CTV was used, based on previous studies,^7^, ^8^, ^9^ and the motion evaluation based on the 3 breath-hold scans from the simulation. Within our current patient cohort, a 5 mm margin was used for 9 patients, and a 7 mm margin was used for 1 patient.

A preplan was created using our planning template, prioritizing target coverage objectives and OAR constraints. During the treatment of each fraction, a CBCT scan was performed while the patient held their breath. The adaptation process involved 3 major steps: influencer generation and review, target and OAR generation and review, and treatment plan generation, review, and QA.^17^ Based on AI techniques, the oART system identified OARs close to the targets, referred to as influencers. In Ethos, for the treatment site of the stomach, the heart, stomach, liver, bowel, and lungs were listed as the influencers. Therapists then reviewed and refined the contours of these influencers. The influencer contours guided the nonrigid registration to deform the OAR and target shapes. The proposed OAR and target contours were then reviewed by the physician, and the oART system calculated the dose distribution based on the preplan with the new contours, defining this as the scheduled plan. Using contour derivation formulas, constraints, and priorities from the preplan, the oART system also optimized and generated a new adapted plan. Physicians then compared the scheduled and adapted plans, selecting the best plan based on target coverage and OAR sparing. After plan selection, another verification CBCT was performed to ensure minimal motion between the pretreatment and planning CBCT. During treatment, a threshold of 3 mm in the SGRT system was used to manually gate breath-hold beam delivery, ensuring breath-hold consistency.

### Preplan approach

A general planning strategy was developed for patients with gastric MALT lymphoma, although variability existed based on target size and location. The preplan was generated using the physician-contoured CTV and OARs, and the PTV was derived with a 0.5 to 0.7 cm margin expansion of the CTV. Ring structures of 0.5 cm thickness at various distances from the PTV were defined to control the fall-off dose beyond the target. These tuning structures were automatically regenerated during the adaptive session based on the new CTV contour defined by the Doctor of Medicine (MD) on the console on the day of treatment. The planning optimization prioritized CTV and PTV coverage with a hot spot constraint (D0.03 cc < 108%) and then prioritized dose fall-off with the rings using D0.03 cc constraints. It was followed up by constraints on other OARs, which can be at a first- or second-level priority. While the preplan can be prioritized at 4 levels, the console only visualizes first- and second-level priority during treatment. Therefore, most of the PTV and OARs were kept in P1 and P2. Table E1 shows the generalized preplan template. The dose constraints were mainly based on previous publications, as well as the National Comprehensive Cancer Network Hodgkin lymphoma constraints.^2^^,^^18^ Considering patient couch time and comfort, different plans were generated with multiple intensity modulated RT beam arrangements (ie, number of beams, gantry angle, and collimator angle variation) during preplanning. The plan with the least monitor units (MUs) with acceptable quality was chosen. All plans were set to normalize to 95% PTV coverage in the Ethos treatment planning system (Version 1.1).

### Adapted plans evaluation

To evaluate the necessity and efficacy of oART, the coverage of the PTV, CTV, Paddick conformity index (CI),^19^ gradient index (GI), and OAR doses were compared among the preplans, scheduled plans, and adapted plans. The Paddick CI is defined as $\frac{{\left(TV\cap PIV\right)}^{2}}{TV\times PIV}$, where TV is the target volume and PIV is the prescription isodose volume. GI is defined as $\frac{PIV_{0.5}}{PIV},$ where $PIV_{0.5}\;$is defined as the half-prescription isodose volume. Paired 2-sided Wilcoxon tests were used for statistical comparisons. The OAR doses in the adapted plan were also compared with values from the literature with conventional IGRT results.

To evaluate the robustness of oART, the adapted plan MUs, Paddick CI, and GI were compared with the preplan using the percentage relative difference. The percentage relative difference was defined as (Adapted − Preplan)/Preplan × 100%. MUs were reported because they are related to the number of breath-holds across a treatment, and the Paddick CI and GI were reported to evaluate the dose conformity and the dose fallout gradient.

### Treatment efficiency: time statistics

Treatment time is crucial for the oART process because it demonstrates the resources required for each treatment. In this study, we reported the treatment time for each step of the adaptive treatment workflow for 93 fractions from 9 patients because the rest of the data were not available. The time statistics, including influencer generation and review, target and OAR generation and review, treatment plan generation review and QA, beam delivery, adapted workflow, and MD at the console, were summarized in Table 1. The adaptive workflow was defined as the time from the start of influencer generation to the treatment plan approval. After the therapists edited the influencer contours, the MD at console time included the time from target and OAR contour review to treatment plan approval.

**Table 1:** Time statistics for the time spent on each step for the adapted workflow, beam delivery, the total adapted workflow time (from influencer generation to plan approval), and MD on console time (from target and organ at risk [OAR] generation and review to plan approval)

Variable	Mean (min)	Standard deviation (min)	Minimum (min)	Maximum (min)
Influencer generation and review	5.4	3.4	0.7	17.0
Target and OAR generation and review	14.1	5.9	3.8	32.7
Treatment plan generation, review, and QA	4.8	1.3	2.0	11.3
Beam delivery	15.0	4.4	8.0	29.0
Adapted workflow	25.0	6.8	9.5	53.0
MD at console	18.9	6.3	6.6	38.8

Abbreviations: MD = doctor of medicine; QA = quality assurance .

## Results

### Adapted plan evaluation: overview

**Figure 1:**
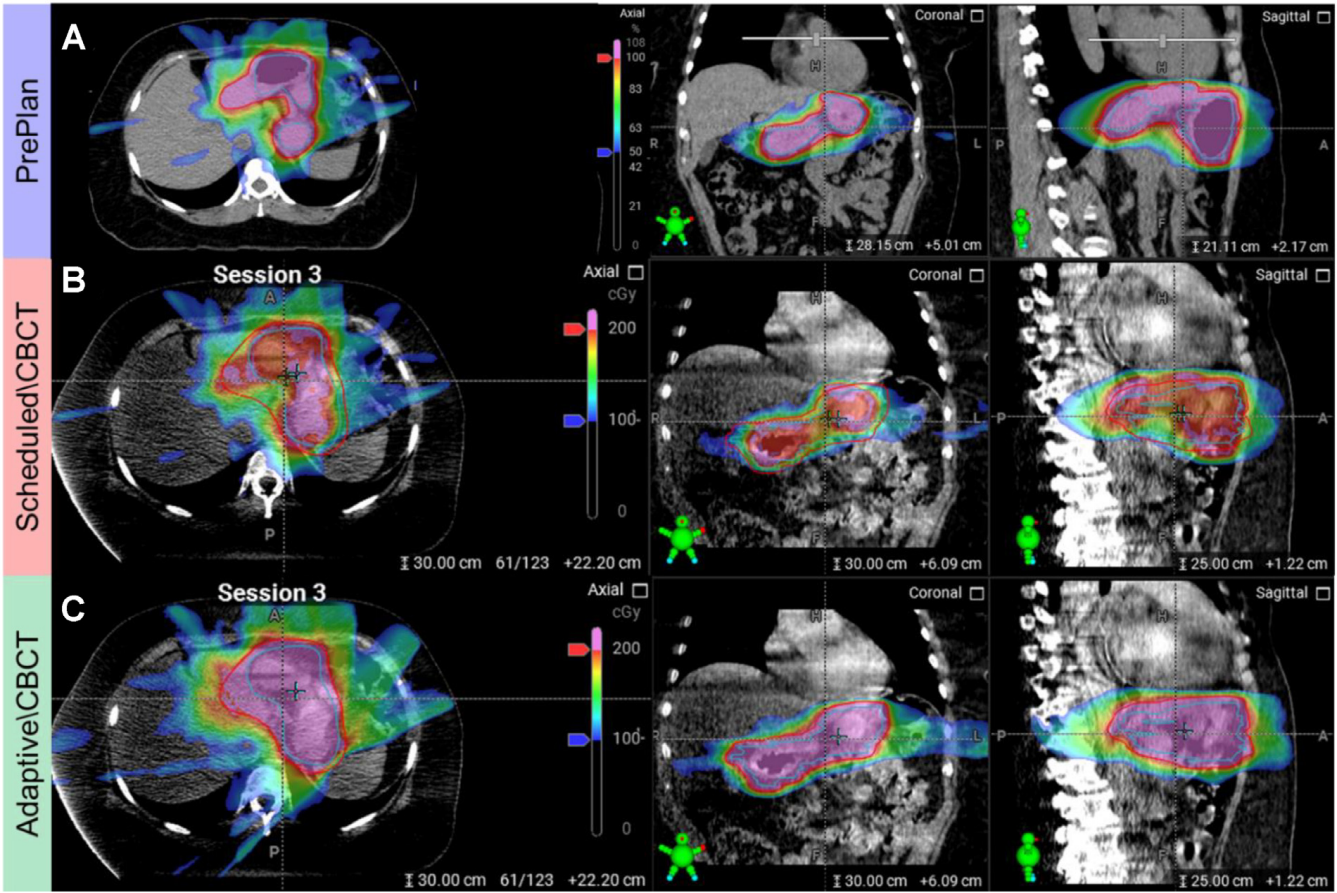
Dose distribution for (A) Preplan from simulation, (B) scheduled plan during an example fraction with cone-beam computed tomography (CBCT), and (C) adapted plan during an example fraction with CBCT. Blue outline and red outline represent CTV and PTV respectively with color wash as the dose distribution. The 100% dose area is marked pink while the rest of the dose wash represents the dose from 50% to 100%. The scheduled plan is preplan calculated on the adapted anatomy. *Abbreviations:* CTV = clinical target volume; PTV = planning target volume.

The adapted plans were selected for every fraction because of superior target coverage and similar OAR sparing. Figure 1 demonstrates an example dose distribution for the preplan, scheduled plan, and adapted plan for the same fraction. The prescription dose per fraction in Fig. 1 was 200 cGy. Blue and red outlines represent the CTV and PTV, respectively, with color wash as the dose distribution. The 100% dose area is marked as pink, while the rest of the dose wash represents the dose from 50% to 100%. While the preplan showed good target volume coverage, the scheduled plan showed undercoverage of the target because of target deformation, and the adapted plan recovered the target coverage with reoptimization.

**Figure 2:**
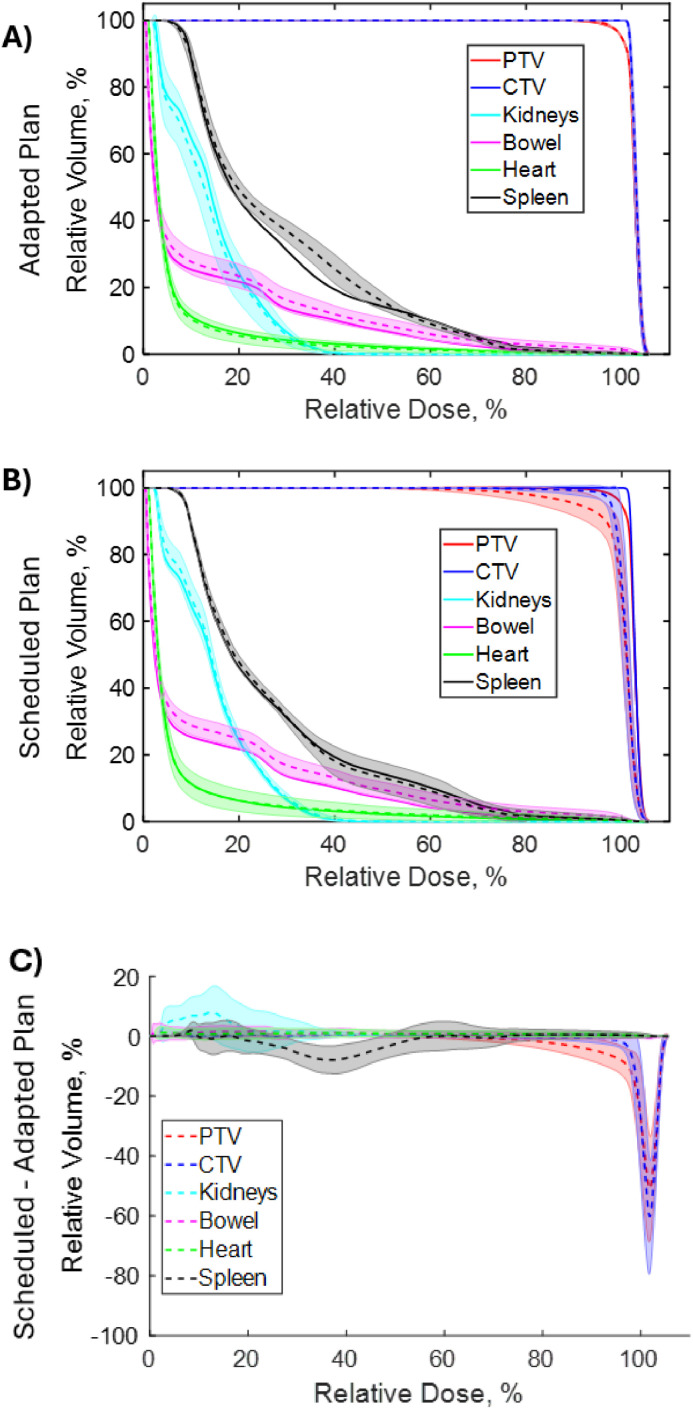
Dose-volume histograms (DVHs) for an example patient, comparing all adapted plans (A), all scheduled plans (B), and the differences between scheduled and adapted plans (C). The solid lines represent the preplan DVHs, while the dashed lines represent the mean DVHs across either the adapted or scheduled plans. Shaded bands indicate the standard deviation of the DVHs across the adapted or scheduled plans.

Similarly, Fig. 2 shows the dose-volume histograms (DVHs) for targets and OARs for 1 example patient. The dashed lines represent the mean DVHs, while the bands show the standard deviation of the DVHs of all adapted plans (A) and scheduled plans (B). The solid lines represent the preplan DVHs. The targets’ DVHs from the adapted plans are nearly identical, whereas the OAR dose has a narrow band, indicating that the adapted plan achieved consistent dosimetry metrics, while the targets’ DVHs from the scheduled plan have wider bands. The adapted plans exhibit lower DVH deviations for the targets while maintaining similar OAR variations compared with the scheduled plans. Figure 2C demonstrates differences in DVHs between the scheduled and adapted plans, with the most significant differences observed in the PTV and CTV.

### Adapted plan evaluation: targets and plan quality

**Figure 3:**
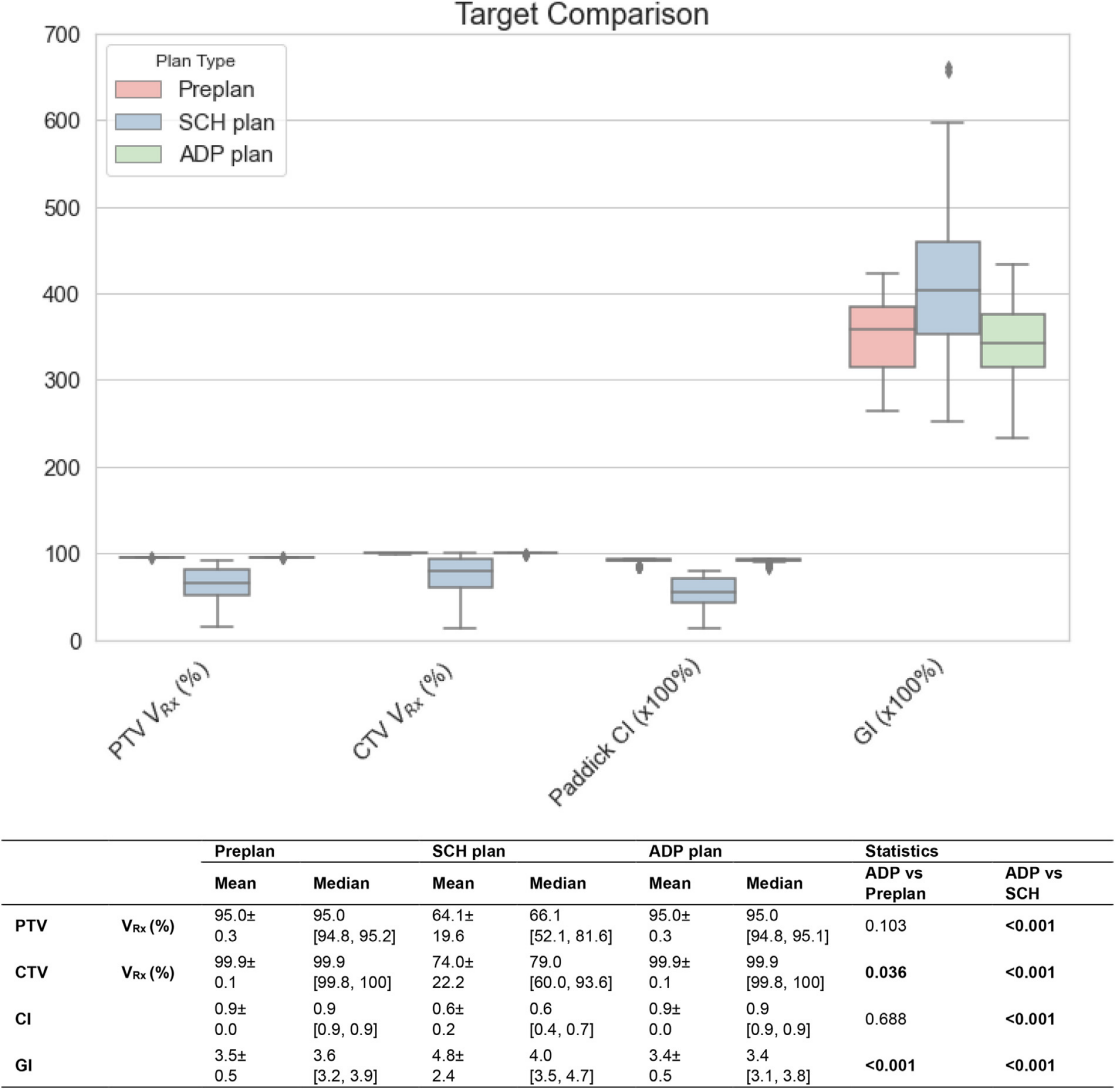
Box and whisker plots comparing planning target volume (PTV) and clinical target volume (CTV) coverage, Paddick conformity index (CI), and gradient Index (GI) across preplans, scheduled plans (SCH plan), and adapted plans (ADP plan) for all fractions (n = 108) from 10 patients. The mean ± standard deviation and median (1st quartile and 3rd quartile) values for each parameter are presented in the accompanying table. Wilcoxon signed-rank tests were performed to compare ADP plans versus preplan, as well as ADP versus SCH plans. Statistically significant *P* values (*P* < .05) are highlighted in bold.

Figure 3 shows the CTV, PTV coverage, Paddick CI, and GI comparison among the preplans, scheduled plans, and adapted plans for all patients. All parameters are significantly different between the adapted and scheduled plans (*P* < .001). The PTV and CTV coverage was V_Rx_ = 95.0% ± 0.2% versus 64.1 ± 19.6% and V_Rx_ = 99.9 ± 0.1% versus 74.0% ± 22.2%, respectively, between the adapted and scheduled plan. The Paddick CI was 0.9 ± 0.0 for the adapted plans and 0.6 ± 0.2 for the scheduled plans. A relative PTV volume change of 11.7% ± 18.5%, with a range from −37.1% to 90.5%, was observed between the adapted plans and preplan, with no apparent trend of volume increase or decrease across fractions. This significant volume variation indicates that the coverage and conformity drop in the scheduled plan was mainly because of significant shifts and deformations of the target for each fraction.

The adapted plan quality was also compared with the preplan regarding MU, GI, and Paddick CI to evaluate robustness, with the relative percentage differences reported. The relative percentage differences of the adapted plans’ MU, GI, and Paddick CI were 4.1% ± 10.4%, −4.2% ± 6.4%, and 0.4% ± 1.6% of the preplan values, respectively. A decreased MU, a decreased GI, or an increased Paddick CI indicates a better plan.

### Adapted plan evaluation: OARs

**Figure 4:**
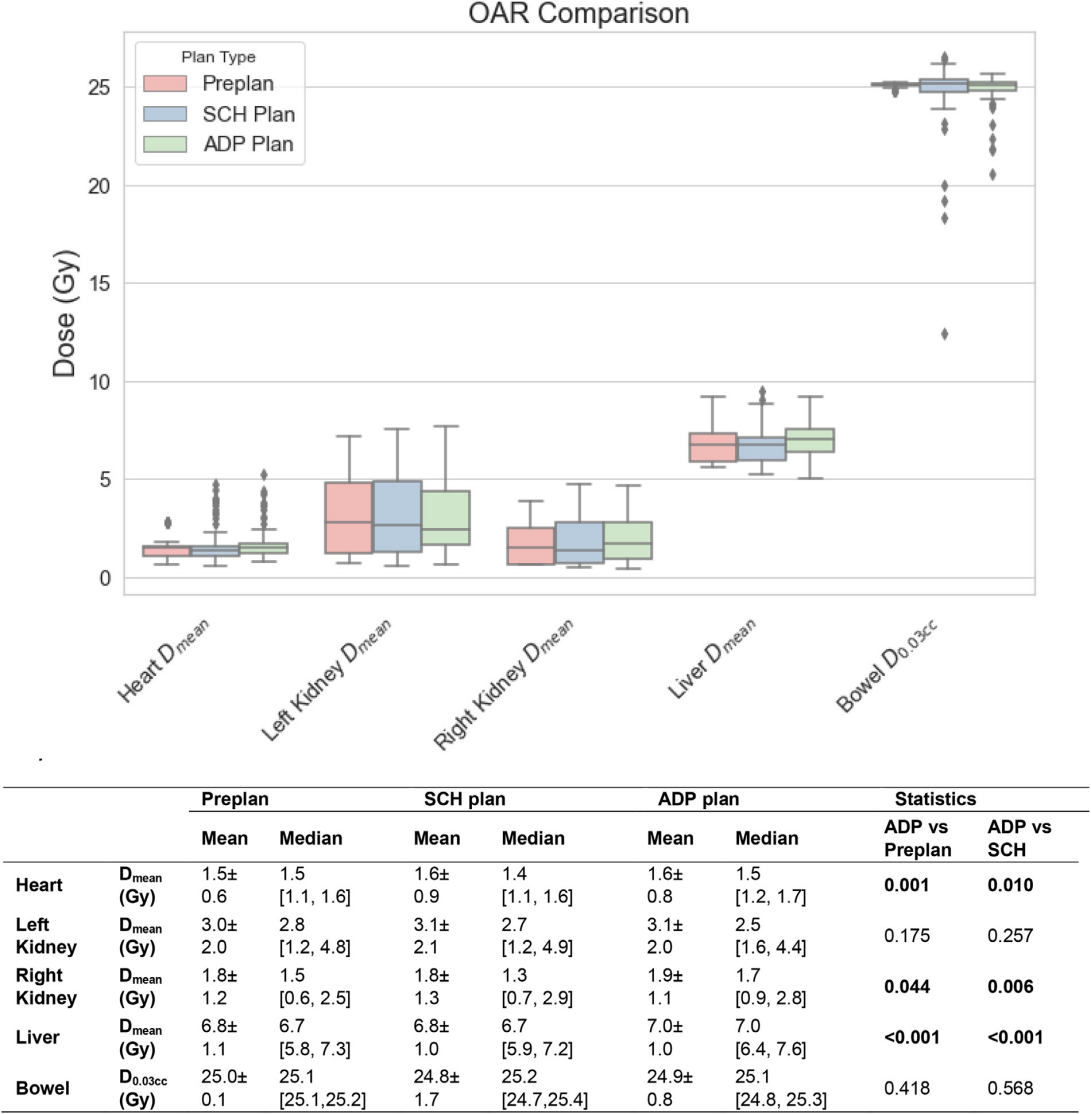
Box and whisker plots showing the mean doses for the heart, left kidney, right kidney, liver, and the D_0.03cc_ for the bowel, comparing the preplan, scheduled plans (SCH plan), and adapted plans (ADP plan) for all fractions (n = 106) from 9 patients. The patient receiving a prescription of 4 Gy in 2 fractions was excluded from this analysis. The mean ± standard deviation and median (1st quartile and 3rd quartile) values for each parameter are provided in the accompanying table. Wilcoxon signed-rank tests were conducted to compare ADP plans versus preplans, as well as ADP versus SCH plans. Statistically significant *P* values (*P* < .05) are highlighted in bold. *Abbreviations:* OARs = organs at risk.

Figure 4 shows a comparison of OAR doses among the preplans, scheduled plans, and adapted plans. All OAR doses are similar among the 3 plan groups, and the median dose difference for all OARs is within 0.3 Gy. The patient who received a treatment of 4 Gy in 2 fractions with a 7 mm PTV margin was excluded from the OAR dose results, ensuring that all patients included in the results had a 24 Gy prescription and 5 mm PTV margin.

The mean dose for the heart from the adapted plans of 9 patients was 1.6 ± 0.8 Gy, and it was lower than the mean heart dose reported in the conventional IGRT treatment from the previous study, which was 4.1 ± 2.0 Gy.^1^ Similarly, the mean doses to the left kidney, right kidney, and liver from the adapted plans were 3.1 ± 2.0 Gy, 1.9 ± 1.1 Gy, and 7.0 ± 9.6 Gy, respectively, which were lower than the corresponding doses reported in the literature of 5.1 ± 3.5 Gy, 2.6 ± 2.9 Gy, and 8.8 ± 2.9 Gy*.*^1^

### Treatment time summary

Table 1 summarizes the time statistics for the oART treatments. In the 3 major steps, influencer generation and review took 5.4 ± 3.4 minutes, target and OAR generation and review took 14.1 ± 5.9 minutes, and treatment plan generation, review, and QA took 4.8 ± 1.3 minutes. The breath-hold treatment delivery time took an average of 15 minutes. The adaptive workflow and MD at console time were 25.0 ± 6.8 and 18.9 ± 6.3 minutes, respectively. Ninety-four percent of the 93 fractions had an adaptive workflow time within 35 minutes. Each patient was scheduled for a 1-hour slot to account for setup, adapted workflow, and beam delivery.

## Discussion

This study demonstrates that CBCT-based oART can ensure target coverage and consistent sparing of surrounding tissue with reduced PTV margins compared with IGRT for patients with gastric MALT lymphoma. As shown in Fig. 1, significant interfractional motions cause the scheduled plan to have reduced target coverage. Figure 3 further illustrates that the scheduled plan exhibits large CTV and PTV coverage variability. Similarly, other patients with GI cancer with abdominal malignancies may benefit from daily oART when significant target deformation occurs. The results of the target coverage and heart dose from the adapted plans indicate that the main advantages of oART are preserving target coverage and reducing heart dose with reduced PTV margins compared with conventional IGRT. This finding is consistent with the conclusion reported from MRgRT studies.^7^, ^8^, ^9^

**Figure 5:**
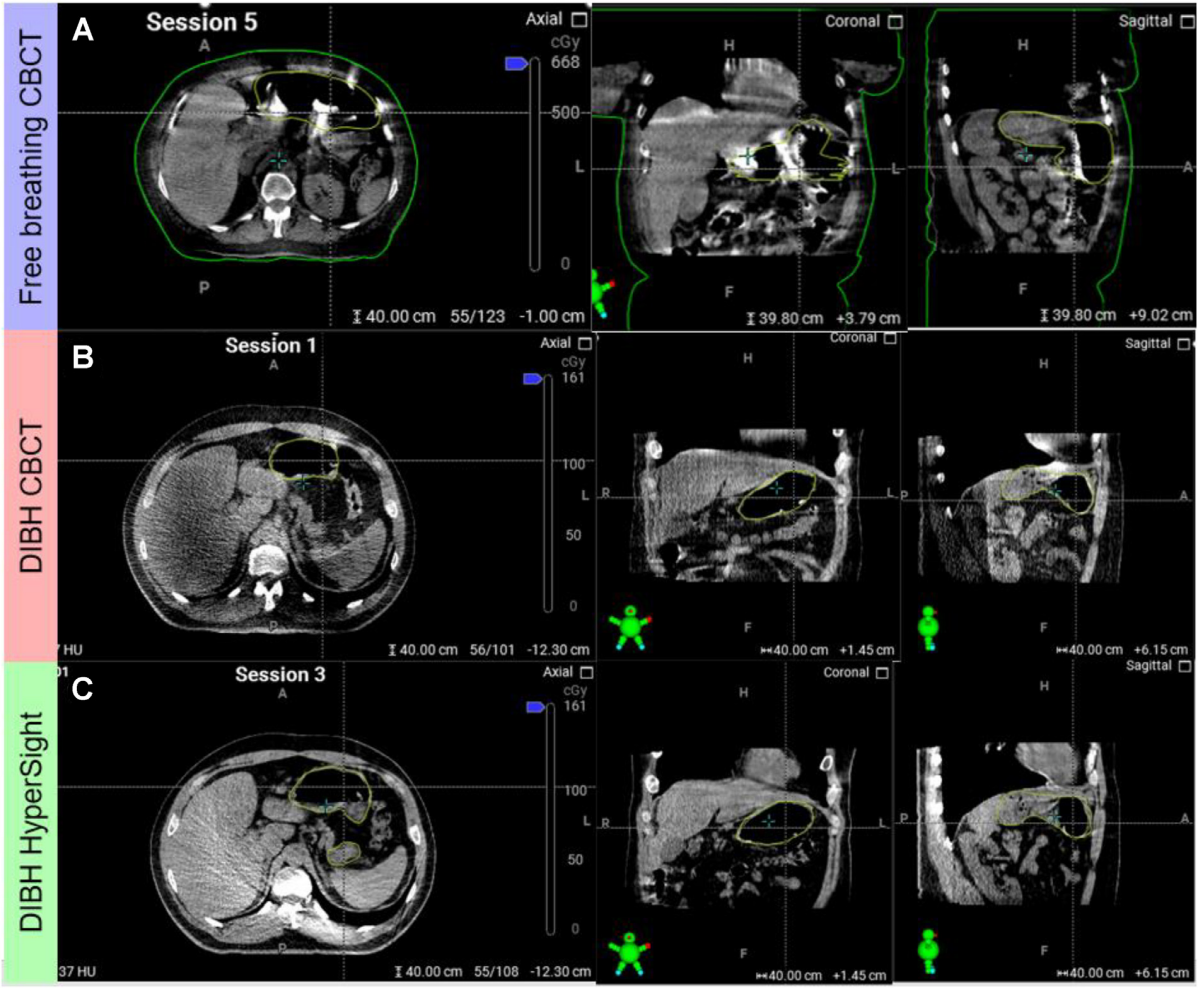
Example cone-beam computed tomography (CBCT) image quality for a free-breathing abdomen patient (a) and another patient with mucosa-associated lymphoid tissue lymphoma treated with conventional CBCT (b) and HyperSight (c) with surface guided radiation therapy deep inspiration breath-hold (DIBH). The green contour represents the stomach (clinical target volume) contour.

It is also important to note that CBCT-based delineations of structures may be more challenging on the day of treatment than compared with MRgRT. As shown in Fig. 1, the image quality of the CBCT on the day of treatment is inferior to that of the simulation CT. However, our experience is that with DIBH and the proposed contours from oART, the image quality is sufficient for physicians to visualize the target and OARs. Figure 5a, b demonstrates that DIBH significantly improves CBCT image quality, and the automatic influencer contours from the oART system generally provide accurate stomach contours requiring only minor modifications. The image quality could be further enhanced by using oral contrast and improved CBCT imaging technologies.^1^ Because daily oral contrast is impractical for patients with many fractions, especially with the stomach as the treatment site, it was not used in the treatments. Regarding imaging techniques, we treated some fractions of a patient with an advanced CBCT platform (HyperSight, Varian Medical Systems^20^) and others with conventional CBCT. With DIBH, either conventional CBCT or HyperSight offered sufficient image quality for visualizing the target and OARs. HyperSight images provided better contrast, as shown in Fig. 5b, c.

The proposed workflow is streamlined to ensure that the adaptive treatment times are reasonable, typically less than 35 minutes. The relatively short adaptive workflow time is partly because of the auto-contouring capabilities of the Ethos system and the short acquisition time of CBCTs.^21^ Consequently, the time physicians spent at the console was kept on average to approximately 19 minutes, including waiting time for their arrival, so the actual MD at console time could be less than reported. Online contouring could be further reduced by integrating the technique to focus on the OAR area close to the target. The MU number from all adapted plans is 1635 ± 313, allowing most patients to complete each treatment within 5 breath-holds. This relatively short treatment time and fewer breath-holds ensure patient comfort and minimize intrafraction motion.

This study has several limitations. First, the number of cases included in the study is small because of the low occurrence of gastric MALT lymphoma at a single institution. However, this is a study with the most patients reported using the oART systems, including MRgRT. Second, the current PTV margin is based on evaluating multiple breath-hold images from the simulation and literature. Intrafraction motions were partially confirmed by the second position verification CBCT, and more quantitative validation of the intrafraction motion will be included in a future study to better determine the optimal PTV margin. Third, with workflow development, treatments are not entirely consistent across all patients. For example, the prescription is different for one of the patients from the other patients because of advanced disease. However, this should not affect our conclusion on the feasibility and effectiveness of the CBCT guided oART workflow for gastric MALT lymphoma treatment. During data analysis, comparisons were made mainly within each patient to ensure a fair comparison, and the patient receiving 4 Gy with the 7 mm PTV margin was excluded from the OAR dose statistics. Fourth, the Ethos 1.0 treatment planning system currently uses synthetic CT generated by deforming the simulation CT to align with CBCT anatomy. As a result, tissue inhomogeneities, such as gas, are not accurately represented in the synthetic CT, contributing to dose calculation uncertainties. Users should carefully review the CBCT before proceeding with adaptive workflow to minimize uncertainty. However, with advancements such as the HyperSight CBCT technique, direct CBCT-based dose calculation could be employed in the future, thereby reducing these uncertainties.

## Conclusion

This study presents a streamlined strategy for gastric MALT lymphoma treatments using a CBCT-based oART system. The adapted plans ensure target coverage with a reduced PTV margin that allows better sparing of nearby OARs. The workflows and plans achieve a reasonable adaptive workflow time for most patients, typically less than 35 minutes, facilitating the adaptation of CBCT-based daily adaptive RT for patients with gastric MALT lymphoma. These strategies are not unique to gastric MALT lymphoma and could benefit other malignancies of gastrointestinal and abdominal sites.

## Appendix Supplementary materials

SupplementalMaterial_new Image, application 1

## Footnotes

Supplementary material associated with this article can be found in the online version at doi:10.1016/j.adro.2024.101692.
