# Supplementary material for: Cone Beam Online Adaptive Radiation Therapy: A Promising Approach for Gastric Mucosa-Associated Lymphoid Tissue Lymphoma?
Source: Adv Radiat Oncol. 2024 Dec 2;10(2):101692. doi: 10.1016/j.adro.2024.101692 (PMC11733034; doi:10.1016/j.adro.2024.101692)
Supplement: SupplementalMaterial_new [file mmc1.docx]

| Structure (Formula) | Goal | Order (P1, P2, etc) |
| --- | --- | --- |
| PTV | $D_{0.03cc}<108\%Rx$,  $V_{100\%}>95\%Rx$ $D_{99\%}>90\%$ | P1 |
| CTV | $V_{100\%}>99\%Rx$ | P1 |
| Heart | $D_{mean}\leq2Gy (8Gy)$ | P1 |
| Ring_3mm_ | $D_{0.03cc}<Rx$ | P1 |
| Ring_2/3cm_ | $D_{0.03cc}<50\%Rx (67\% Rx)$ | P1 |
| Spleen | $D_{mean}<8Gy(10Gy)$  $V_{5Gy}\leq30\%$  $V_{15Gy}\leq20\%$ | P1-P2 |
| Bowel | $V_{15Gy}\leq120cc$ | P1-P2 |
| Liver | $D_{mean}<14Gy (15Gy)$  $V_{20Gy}\leq30\%$  $V_{30Gy}\leq20\%$ | P1-P2 |
| Kidney | $D_{mean}<6Gy (8Gy)$ | P1-P2 |
| Other OARs | NCCN Hodgkin’s Lymphoma Constraints | P1-P2 |
| Bowel | $D_{0.03cc}<45Gy$ | PR |
|  |  |  |

**Table E1.** General planning strategy for Ethos gastric MALT lymphoma plans. D_0.03cc_ is used to determine maximum dose.

| **Patient** | **Age** | **Stage** | **Rx**  **(Gy(fx))** | **PTV Margin (mm)** | **Initial PTV Volume (cc)** | **BH Motion Management** | **Number of Fields** |
| --- | --- | --- | --- | --- | --- | --- | --- |
| **1** | 60 | IE | 24(12) | 5 | 250.8 | ABC | 12 |
| **2** | 62 | IE | 24(12) | 5 | 350.9 | ABC | 12 |
| **3** | 63 | IE | 24(16) | 5 | 433.9 | ABC | 6 |
| **4** | 60 | IE | 24(12) | 5 | 574.6 | SGRT | 9 |
| **5** | 67 | IVE | 4(2) | 7 | 804.8 | SGRT | 10 |
| **6** | 68 | IE | 24(12) | 5 | 380.3 | SGRT | 15 |
| **7** | 66 | IE | 24(12) | 5 | 878.8 | SGRT | 11 |
| **8** | 60 | IE | 24(12) | 5 | 540.6 | SGRT | 11 |
| **9** | 69 | IE | 24(12) | 5 | 399 | SGRT | 12 |
| **10** | 53 | IE | 24(16) | 5 | 810.5 | SGRT | 12 |

**Table S2.** Patient cohort summary including patient age, disease staging, PTV margin, initial PTV volume, type of motion management and number of fields.
